# Raucedo Catarrhalis Sometimes Mistaken for Croup

**Published:** 1814-05

**Authors:** 


*For the Medical and Physical Journal.*

### *Raucedo Catarrhalis sometimes mistaken for Croup.*

EVERY practitioner of observation will readily admit that Cynanche Trachealis, or Croup, is a disease which, within these few years, has become much more frequent than it used to be. This is probably to be imputed to improper clothing and improper diet, rather than to any difference in regard to the humidity or temperature of the atmosphere, which, taking one season with another, must be considered to be the same now as they were twenty or thirty years ago.

But, although it be true, whatever be the cause, that more cases of Croup occur now than formerly; yet it is by no means quite so common a disease as some persons imagine, who, from haste and want of accurate discernment, mistake the hoarse voice and cough, which sometimes accompany the beginning of a catarrhal attack in young subjects, for symptoms symptoms of croup. This raucedo catarrhalis is easily removed by the common febrifuge treatment; and the deceived practitioner takes to himself the credit of having cured a case of croup.

The mistake arises from attending to sound alone. In the raucedo catarrhalis, the noise resembling the croup sound is produced only in the act of speaking and coughing, but not during inspiration; whereas in cynanche trachealis the peculiar sound is greatest while the air is drawn into the lungs. But there is another circumstance which affords material assistance in establishing a diagnosis between the two affections; I mean the dypsnœa and convulsive action of the parts concerned in respiration. These are symptoms inseparable from croup, but are not present in the raucedo catarrhalis. *A London Practitioner.*

*March* 10, 1814.

